# Fisetin Inhibits Migration and Invasion of Human Cervical Cancer Cells by Down-Regulating Urokinase Plasminogen Activator Expression through Suppressing the p38 MAPK-Dependent NF-κB Signaling Pathway

**DOI:** 10.1371/journal.pone.0071983

**Published:** 2013-08-05

**Authors:** Ruey-Hwang Chou, Shu-Ching Hsieh, Yung-Luen Yu, Min-Hsien Huang, Yi-Chang Huang, Yi-Hsien Hsieh

**Affiliations:** 1 Graduate Institute of Cancer Biology and Center for Molecular Medicine, China Medical University, Taichung, Taiwan; 2 Department of Biotechnology, Asia University, Taichung, Taiwan; 3 Institute of Medicine, Chung Shan Medical University, Taichung, Taiwan; 4 Department of Rehabilitation Science, Department of Acupressure Technology, Jen-Teh Junior College of Medicine, Nursing and Management, Miaoli, County, Taiwan; 5 Institute of Biochemistry and Biotechnology, College of Medicine, Chung Shan Medical University, Taichung, Taiwan; 6 Department of Biochemistry, School of Medicine, Chung Shan Medical University, Taichung, Taiwan; 7 Department of Clinical Laboratory, Chung Shan Medical University Hospital, Taichung, Taiwan; Wayne State University School of Medicine, United States of America

## Abstract

Fisetin (3,3’,4’,7-tetrahydroxyflavone), a naturally occurring flavonoid, has been reported to inhibit proliferation and induce apoptosis in several cancer types. However, its effect on the anti-metastatic potential of cervical cancer cells remains unclear. In the present study, we found that fisetin inhibits the invasion and migration of cervical cancer cells. The expression and activity of urokinase plasminogen activator (uPA) was significantly suppressed by fisetin in a dose-dependent manner. We also demonstrated that fisetin reduces the phosphorylation of p38 MAPK, but not that of ERK1/2, JNK1/2, or AKT. Addition of a p38 MAPK inhibitor, SB203580, further enhanced the inhibitory effect of fisetin on the expression and activity of uPA and the invasion and motility in cervical cancer cells. Fisetin suppressed the TPA (tetradecanoylphorbol-13-acetate)-induced activation of p38 MAPK and uPA, and inhibited the TPA-enhanced migratory and invasive abilities. Furthermore, the promoter activity of the uPA gene was dramatically repressed by fisetin, which disrupted the nuclear translocation of NF-κB and its binding amount on the promoter of the uPA gene, and these suppressive effects could be further enhanced by SB203580. This study provides strong evidence for the molecular mechanism of fisetin in inhibiting the aggressive phenotypes by repression of uPA via interruption of p38 MAPK-dependent NF-κB signaling pathway in cervical cancer cells and thus contributes insight to the potential of using fisetin as a therapeutic strategy against cervical cancer by inhibiting migration and invasion.

## Introduction

Cervical cancer is a leading cause of mortality in women worldwide, and its global incidence increased at an annual rate of 0.6% between 1980 and 2010 [1]. Although cervical cancer death rates have been decreasing, the recurrence and metastasis of cervical carcinoma to other sites such as the lymph nodes [2,3], lungs [4,5], bones [6,7], liver [8], and bowels [9] are critical factors contributing to mortality in cervical cancer patients. Therefore, apart from surgery and the destruction of cervical cancer cells by medication, inhibiting metastasis is an auxiliary strategy for curing patients of cancers.

Herbal medicines have been used to treat a variety of cancers, including leukemia as well as cervical, ovarian, testicular, lung, liver, esophageal, stomach, colon, and rectum cancer [10]. Some herbal medicines, such as garlic, mistletoe, Lingzhi, and astragalus have been reported to possess anticancer and chemopreventive potential [11]. These herbal medicines as well as a variety of other plant species contain polyphenolic compounds known as flavonoids [12,13], which have been shown to possess anticancer and chemopreventive properties through their antioxidant activity and their ability to inhibit proliferation and angiogenesis as well as induce cell-cycle arrest, apoptosis, and differentiation [14]. Increasing evidences indicate that some flavonoids derived from natural products are potent chemopreventive agents with low cytotoxicity [15].

Fisetin (3,3’,4’,7-tetrahydroxyflavone) is a naturally occurring flavonoid commonly found in fruits and vegetables such as apples, persimmons, strawberries, cucumbers, and onions [16]. It exhibits a variety of biological functions, including anti-oxidative [17], anti-inflammatory [18], and anti-proliferative activities [19]. The effects of fisetin against cancer have been demonstrated for several cancer types, including hepatoma [20], promyeloleukemia [21], lung adenocarcinoma [22], and prostate [23]. Fisetin induces apoptosis in various cancer cells through different mechanisms, it inhibits COX2 and Wnt/EGFR/NF-κB in HT-29 human colon cancer cells [24] and activates caspase-3 cascade in SK-HEP-1 hepatocellular carcinoma cells [20] and caspase-3 and Ca^2+^-dependent endonuclease in HL-60 human promyeloleukemic cells [11]. Recently, we found that fisetin also induces apoptotic cell death through the ERK1/2-mediated activation of the caspase-8/caspase-3-dependent pathway in HeLa human cervical adenocarcinoma cells [25]. Previous studies have shown that fisetin also induces autophagic cell death by inhibiting both the mTORC1 and mTORC2 pathways in PC-3 human prostate cancer cells [26]. However, the anti-metastatic property of fisetin has not been well documented.

Cancer metastasis is the leading cause of poor clinical outcomes and mortality in cancer patients. The metastatic process involves cell adhesion, migration, invasion, as well as proteolytic degradation of the extracellular matrix (ECM) [27]. Degradation of ECM components is a critical step in the metastatic process, and it is regulated by the activation of proteases, such as urokinase plasminogen activator (uPA) [28] and matrix metalloproteinases (MMPs) [29]. Urokinase plasminogen activator converses the inactive zymogen plasminogen by proteolytic cleavage to activate the serine proteinase plasmin, which in turn catalyzes the degradation of ECM, thereby facilitating the invasion of cancer cells. The uPA cascade consists of uPA, the uPA receptor (uPAR), plasminogen, and plasmin, and the dysregulation of the uPA/plasmin network affects cancer malignancy. The transcription of the uPA gene is known to be regulated by binding NF-κB on its promoter [30]. NF-κB is a heterodimeric transcription factor composed of an REL family/p65 and p50 or p52 subunits. After dissociating from the inhibitor of NF-κB (IκB) in the cytoplasm, NF-κB translocates into the nucleus and activates its target gene to promote the proliferation and metastasis of cancer cells [31]. Therefore, in this study, we investigated the effects of fisetin on cell invasion and its related signaling pathway in cervical cancer cells.

In brief, we demonstrated that fisetin inhibits the phosphorylation of p38 MAPK and disrupts the nuclear translocation of NF-κB to reduce the expression of uPA, thereby suppressing the migration and invasion of human cervical cancer cells. This study provides insight on the potential of fisetin to inhibit metastasis in the treatment of cervical cancer.

## Materials and Methods

### Reagents and Antibodies

Fisetin (3,3,4,7-tetrahydroxyflavone) was purchased from Sigma (St. Louis, MO). Stock solution of fisetin was prepared at 100 mM in DMSO and stored at -20 °C. MTT [3-(4,5-dimethylthiazol-2-yl)-2,5-diphenyltetrazolium bromide], DAPI (4’-6-diamidino-2-phenylindole) were purchased from Sigma (St. Louis, MO). SB203580 and TPA (12-O-tetradecanoylphorbol-13-acetate) were bought from Calbiochem (San Diego, CA). The antibodies against p-ERK1/2, ERK1/2, p-p38 MAPK, p38 MAPK, p-JNK, JNK1/2, uPA, α-tubulin, NF-κB (p65) and β-actin were purchased from Santa Cruz Biotechnology (Santa Cruz, CA). Lamin B was purchased from Millipore (Darmstadt, Germany). Horseradish peroxidase-conjugated anti-mouse and anti-rabbit secondary antibodies were obtained from Promega (Madison, WI). The p38 MAPK inhibitor SB203580 was purchased from Calbiochem (San Diego, CA). The uPA promoter constructs cloned into the pGL3-Basic luciferase vector (Promega) and β-galactosidase plasmids were donated by Dr. JL Ko of the Institute of Medicine, Chung Shan Medical University, Taichung, Taiwan

### Cell Culture

Human cervical adenocarcinoma SiHa and CaSki cells were obtained from the American Type Culture Collection (Rockville, MD, USA). The SiHa cells were maintained in DMEM medium and CaSki cells were maintained in RPMI-1640 medium, these cells were supplemented with supplemented with 2 mM glutamine, 100 U/ml penicillin and 100 µg/ml streptomycin (Sigma), and 10% heat-inactivated fetal bovine serum (FBS; HyClone, Logan, UT). The cultures were incubated at 37°C in a humidified atmosphere with 5% CO2. Cells were passaged every 2 days to obtain an exponential growth.

### Cell Viability Assay

The cell viability was determined by MTT assay. Cells were seeded at a density of 3 ×10^4^ cells/well in a 24-well plate and cultured for 24 h. The cells were treated with various concentrations of fisetin for 24 or 48 h. Subsequently, the medium was replaced with fresh medium containing 0.5 mg/ml MTT for 4 h. The number of viable cells was proportional to the amount of the reduction of MTT, formazan, by dehydrogenases in the mitochondria within live cells. The medium was removed and the produced formazan was dissolved in isopropanol and measured at 570 nm by a Multiskan MS ELSA reader (Labsystems, Helsinki, Finland). The relative cell number was normalized by the absorbance from the untreated cells.

### Migration and Invasion Assays

Cell migration and invasion assay was performed as described previously with modifications [32]. For the migration assay, the cells (2×10^5^ cells/well) were treated with fisetin (0, 10, 20, and 40 µM) for 48 h, then trypsinized and resuspended in serum-free medium and 5×10^4^ cells were placed in the upper chamber of the well insert with 8 µm pore size polycarbonate membrane filter (Millipore). DMEM containing 20% fetal bovine serum was placed in the lower chamber. For the invasion assay, the experimental procedures are similar to the migration assay as described above, except the well insert was coated with 10 µL Matrigel (5 mg/mL; BD Biosciences, Bedford, MA) (50 µg/well) to mimic the ECM barrier before use. After incubation for 12 h or 24 h (SiHa cells) and for 36 and 48 h (CaSki cells) at 37°C in the migration or invasion assay, respectively, the cells on the upper surface of the membrane were removed by cotton swab, The migrated or invaded cells on the lower surface of the membrane were fixed with methanol and stained with 0.05% Giemsa, and the cells were counted under a light microscope at 200X magnification. This experiment was performed twice independently. The data are presented as means ± standard deviation of 5 fields from each well of triplicate samples

### Detection of uPA Activity by Casein Zymography

The uPA activity was examined by casein zymography. The SiHa and CaSki cells (2×10^5^/wells) cells were plated in 6 cm dishes and treated to 0, 10, 20, and 40 µM of fisetin for 48 h. The conditioned medium was then collected. The medium was separated by electrophoresis on 10% sodium dodecyl sulfate polyacrylamide gel electrophoresis containing 0.1% casein and then the gels were soaked in 2.5% Triton X-100 in ddH2O twice for a total of 60 min at room temperature, and incubated in substrate buffer (50 mmol/L of Tris–HCl, 5 mmol/L of CaCl_2_, 0.02% NaN_3_ and 1% triton X-100, pH 8.0) at 37°C for 18 h. Bands corresponding to uPA activity were visualized by negative staining using 0.3% Coomassie blue in 50% methanol and 10% acetic acid.

### RNA Isolation and Reverse Transcriptase Polymerase Chain Reaction (RT-PCR)

Total RNA was extracted with TRIZOL reagent (Invitrogen, Carlsbad, CA) according to the manufacturer’s instruction. Total RNA (2µg) was reverse transcribed to complementary DNA (cDNA) in a reaction mixture containing 2.5 µM oligo (dT) primer, 0.5 mM dNTP mixture, 200 U SuperScript III reverse transcriptase, 40 U RNaseOUT, an RNase inhibitor (all from Invitrogen) and incubated at 50 °C for 50 min. After incubation, the reaction mixture was heat inactivated at 85° C for 5 min and then treated with 2 U RNase H at 37° C for 20 min. The PCR was performed in a reaction mixture containing 2 µl cDNA, 0.2 mM dNTP mixture, 2 µM of each primers, 1 U Taq DNA polymerase, and 1-fold concentration of Thermal Pol Buffer (New England BioLabs, MA, USA) by denaturation at 95 °C for 5 min, followed by amplification of indicated cycles of 95 °C for 30 sec, 54 °C for 30 sec, and 72° C for 30 sec. The specific primer sequences for these genes are as following: uPA: 5'- TTGCGGCCATCTACAGGAG-3’ (forward), 5'-ACTGGGGATCGTTATACATC -3' (reverse), and β-actin: 5'-GCACTCTTCCAGCCTTCCTTCC-3' (forward), 5’- TCACCTTCACCGTTCCAGTTTTT -3’ (reverse). Then, 10 µL of each PCR product were run on a 1.5% agarose gel and bands were visualized under UV. β-actin primers were used as internal control and equal loading.

### Preparation of Whole Cell Lysate and Fractionated Extracts

The whole cell lysate was prepared by lysing the cells in RIPA Buffer (50 mM Tris at pH 7.5, 150 mM NaCl, 1 mM EDTA, 0.25% Na-deoxycholate, 1% NP-40, 1 mM NaF, 1 mM Na _3_VO_4_, 1 mM PMSF, 1 µg/ml aprotinin) by sonication. The soluble extraction was collected from the supernatant after centrifugation at 15000 g for 10 min. The cytosolic and nuclear fractions were extracted as followings: cells were lysed in buffer A (20 mM HEPES at pH 7, 10 mM KCl, 2 mM MgCl_2_, 0.5% NP-40, 1 mM NaF, 1 mM Na _3_VO_4_, 1 mM PMSF, 1 µg/ml aprotinin) on ice, ground in a glass dounce homogenizer, and centrifuged at 1500 g for 10 min. The supernatant is the cytosolic fraction. The nuclear pellet was isolated and washed. The nuclei were lysed in NETN buffer (20 mM Tris at pH 8.0, 150 mM NaCl, 1 mM EDTA, 0.5% NP-40, 1 mM NaF, 1 mM Na _3_VO_4_, 1 mM PMSF, 1 µg/ml aprotinin) by sonication and centrifuged at 12000 g for 20 min.

### Western Blotting

Western blots were performed as described before [33]. Equal amounts of protein extracts were separated by 10 or 12.5% SDS-PAGE and transferred onto a polyvinylidene fluoride (PVDF) membrane (Millipore, Belford, MA). After blocking, the membrane was hybridized with the primary antibody against uPA, NF-κB (p65), Lamin B, ERK-1/2, phosphorylated ERK-1/2, p38 MAPK, phosphorylated-p38 MAPK, JNK-1/2, phosphorylated JNK-1/2, AKT, phosphorylated AKT, β-actin, or α-tubulin at 4^o^C overnight. After washing, the membrane was incubated with HRP-conjugated anti-mouse, anti-goat or anti-rabbit antibody at room temperature for 2 h. Subsequently, proteins were visualized by addition of HRP substrate with enhanced chemiluminescence and detected.

### Immunofluorescence Assay

Cells were seeded on an 8 well Lab-Tek Chambered coverglass (Thermo, Rochester, NY). The next day, media were replaced with or without 20 µM fisetin and cultured for 48 h. After removing the chamber, slides were rinsed with phosphate-buffered saline and cells were fixed with 4% paraformaldehyde and permeabilized in methanol. After washing with phosphate-buffered saline, slides were blocked with 2% bovine serum albumin. Primary and secondary antibodies were incubated in 5% bovine serum albumin. DAPI reagent (Invitrogen) was used as mounting and counterstaining media. The cells were examined and photographed by immunofluorescence microscopy.

### Promoter Activity Assay

The cells were co-transfected with 1 µg of pGL3-uPA luciferase reporter constructs and 1 µg of β-galactosidase reporter plasmid by the Lipofectamine 2000 transfection reagent, followed by fisetin treatment for 48 h. Luciferase activities and β-galactosidase activity were assayed by using the luciferase and β-galactosidase enzyme assay system (Promega). Luciferase activity was normalized with the β-galactosidase activity in cell lysate and calculated as an average of three independent experiments were preformed

### Electrophoretic Mobility Shift Assay (EMSA)

Preparation of nuclear extracts from SiHa cells treated with various concentration of fisetin (10, 20 and 40 µM) for 48 h. Briefly, cells were harvested in ice-cold PBS supplemented with protease inhibitors, then lysed in lysis buffer and 1% Triton X-100. After collection of the cytoplasmic fraction, nuclei were lysed and the nuclear extract proteins were solubilized in NETN buffer supplemented with 10 mM DTT, and protease inhibitor. The EMSA protocol followed the LightShift Chemiluminescent EMSA Kit (Pierce, Rockford, IL) instructions. Double- stranded oligonucleotides containing the sequences corresponding to NF-κB consensus site (5’-AGTTGAGGGGACTTTCCCAGGC-3’, 3’-TCAACTCCCCTGAAAGGGTCC G-5’) were 3’-end labelled with biotin. Binding reactions were carried out in a final volume of 20 µL containing 1 µM of digoxigenin-labelled double-stranded NF-κB, 10 µg of nuclear extract, 1 µg of poly dI/dC and binding buffer [20 mM HEPES, pH 7.6, 1 mM EDTA, 10 mM (NH_4_)_2_SO_4_, 1 mM DTT, 2% Tween 20 and 30 mM KCl]. The mixtures were incubated for 20 min at room temperature. Samples were subjected to electrophoresis in 6% nondenaturating polyacrylamide gel in a 0.5×TBE buffer system. Then DNA and membrane were cross-linked using a UV-light cross-linker instrument. Detection of p65-DNA complexes was performed using the Chemiluminescent Nucleic Acid Detection Module (Pierce Chemical). The unlabeled oligos of NF-κB at 200× were added to compete specifically with labeled oligo binding in the competitive EMSA.

### Chromatin Immunoprecipitation (ChIP) Assay

ChIP assay was performed as previously method [34]. In brief, chromatin and proteins from approximate 2 x 10^6^ cells were crosslinked with 1% formaldehyde for 10 min at room temperature. These cells were collected, lysed, and sonicated on ice to shear the chromatin DNA to a length between 200 bp and 1000 bp using Sonicator 3000 (Misonix, NY, USA). The sonicated chromatin lysate was immunoprecipitated with anti-NF-κB (p65) antibody, and collected with Protein A/G agarose beads (Pierce, IL, USA). The protein/DNA crosslinks of the immunoprecipitated complexes were reversed by incubation in 0.2 M NaCl at 65 °C for 4 h, and then the DNA was purified and applied to PCR as described above to determine the binding ability of p65 to uPA promoter. The sequences of the primers specific to the promoter of uPA gene are 5’- AGCATGACAGCCTCCAGCCAAGTA-3’ (forward), and 5’- ACGTGACCAGAACATAAACAGAGA -3’ (reverse).

### Statistical Analysis

The results were presented as mean ± standard error (S.E.) from three independent experiments. Statistical differences in values were analyzed by Student’s t-test for unpaired data and one-way ANOVA by Instat software (GraphPad Prism4, San Diego, CA) with threshold for significance set at P < 0.05.

## Results

Fisetin Efficiently Suppresses the Migratory and Invasive Abilities of Cervical Cancer Cells under Non-toxic Concentrations.

**Figure 1:**
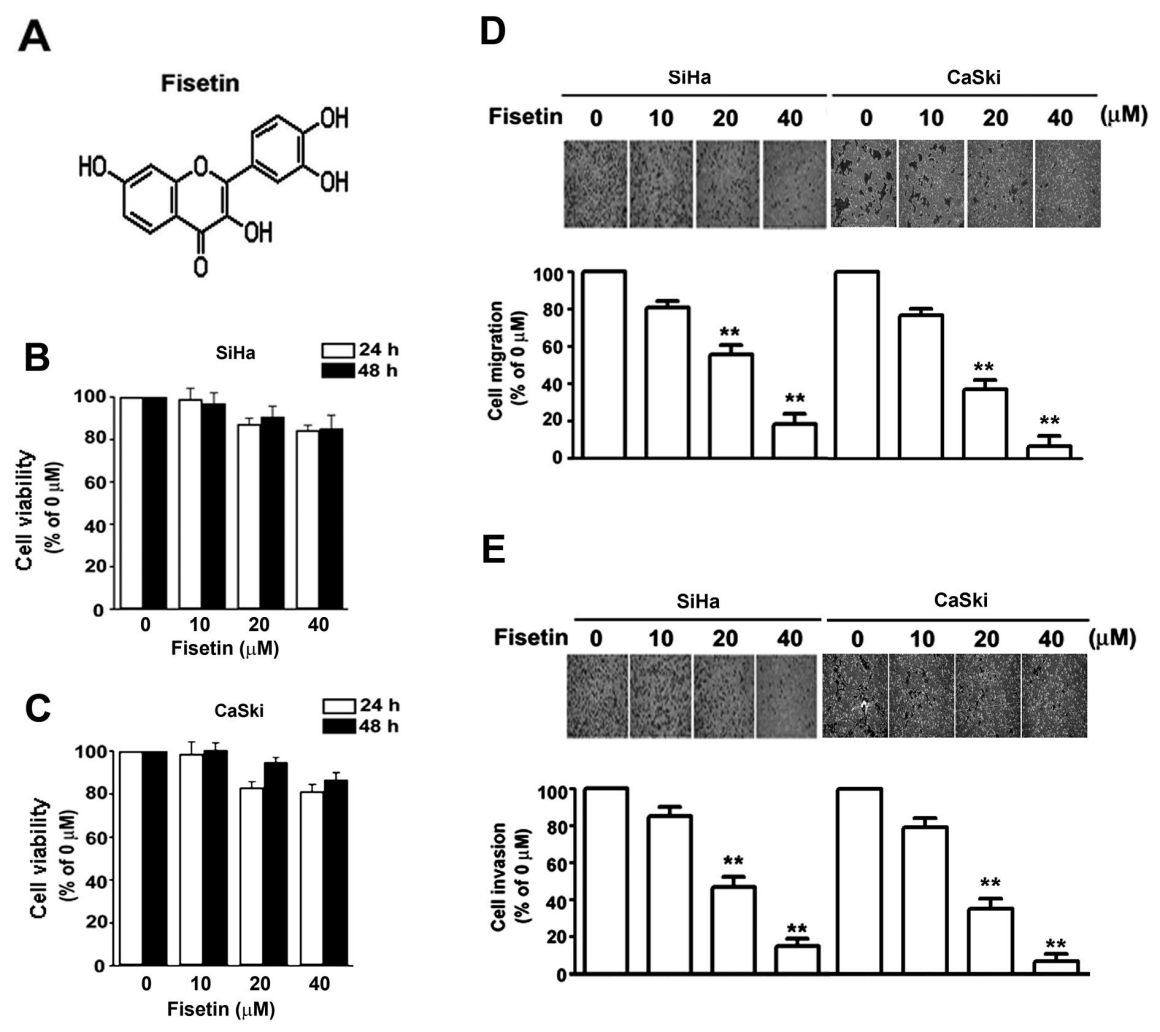
Effects of fisetin on the viability, migration, and invasion in cervical cancer cells. (A) The chemical structure of fisetin. (B) SiHa cells and (C) CaSki cells were treated with increasing concentrations of fisetin for 24 and 48 h. Cell viability was determined by MTT assay. The plot presents relative cell viability compared to untreated (0 µM) cells. SiHa and CaSki cells were treated with the indicated concentrations of fisetin for 48 h. Subsequently, the migratory (D) and invasive (E) ability of cells after each treatment were determined, as described in material and methods. The bottom plots were the relative cell numbers comparing to that in untreated (0 µM) cells. The bars show the value as mean ± S.E. from three independent experiments. **, *P < 0.01*. compared with the untreated cells.

The chemical structure of fisetin is shown in Figure 1A. In this study, we investigated the cytotoxicity of fisetin by treating cervical cancer cells with various concentrations of fisetin for 24 or 48 h. Results from the MTT assay showed that fisetin was not significantly toxic to SiHa (Figure 1B) and CaSki (Figure 1C) cells at the concentrations up to 40 µM for 24 to 48 h. This range of concentrations was therefore applied in all subsequent experiments. Tumor cells detach from neighboring cells by releasing their intercellular junctions during metastasis, and the extracellular-matrix is proteolytically degraded to allow the migration and invasion of cancer cells [35]. To investigate the effect of fisetin on the malignancy of cervical cancer cells, the migration and invasion abilities of SiHa and CaSki cells were determined. In the cell migration assay, SiHa cells treated with 20 and 40 µM fisetin showed a decrease in motility of 46.0% and 81.3%, respectively, and similar result was also observed in CaSki cells with 62.1% and 90.2% of inhibition (Figure 1D). In the cell invasion assay using a Matrigel-coated Boyden chamber, fisetin was shown to reduce cell invasion in a concentration-dependent manner. At 20 µM, invasion was reduced by 52.4% and 59.4%, and at 40 µM, invasion was reduced by 87.2% and 92.4% in SiHa and CaSki cells, respectively (Figure 1E). These results showed that fisetin significantly inhibited the migration and invasion of cervical cancer cells under non-toxic concentrations.

### Fisetin Inhibits the Expression and Activity of uPA in Cervical Cancer Cells

**Figure 2:**
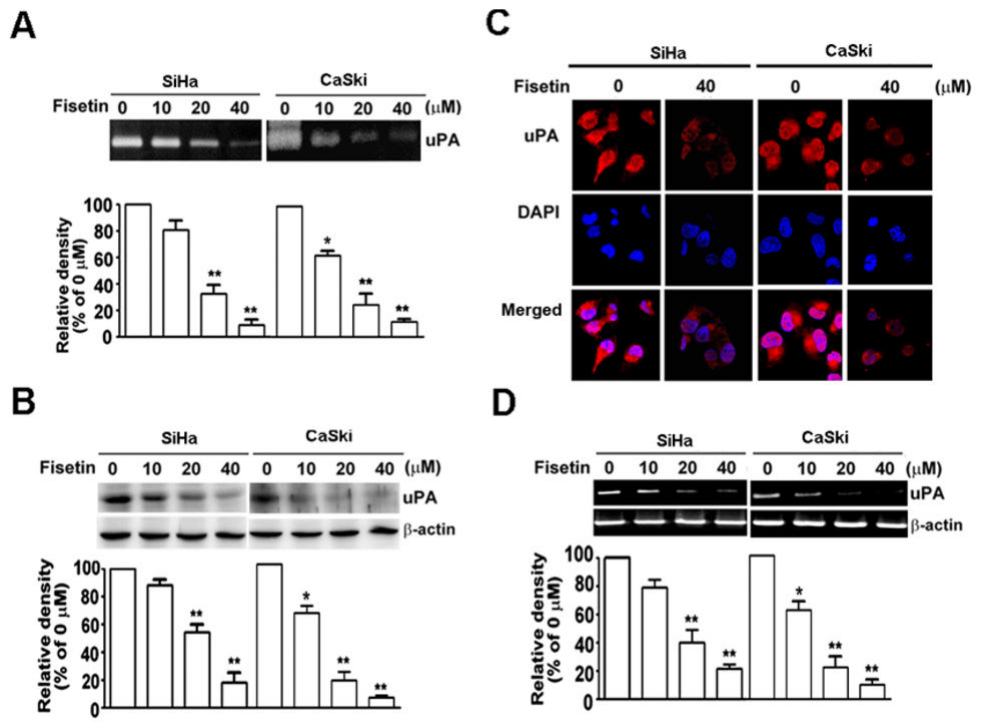
Effects of fisetin on the expression and activity of uPA in cervical cancer cells. Cells were treated with the indicated concentrations of fisetin for 48 h. (A) the conditioned medium from each treatment was collected, and uPA activity was determined by casein zymography. (B) Cell lysate was applied to determine the protein levels of uPA by Western blotting. β-actin was used as the internal control. (C) Cells were fixed, permeabilized, and immunostained with anti-uPA antibody (red), and cell nuclei were counter-stained with DAPI reagent. (D) Total RNA was extracted from each treatment, and the mRNA levels of uPA were examined by RT-PCR. Bars show the value as mean ± S.E. from three independent experiments. *, *P < 0.05*, **, *P < 0.01*, compared with the untreated cells.

Previous studies have shown that an increase in uPA expression is associated with cervical cancer progression [36]. To investigate the possible underlying anti-metastatic effect of fisetin in cervical cancer cells, the activity and expression of uPA in cervical cancer cells treated with various concentrations of fisetin were examined. As shown in the caseinolytic activity assay, uPA activity decreased in a dose-dependent manner after treatment with fisetin. Quantification analysis indicated that uPA activity decreased by 17.2%, 62.3%, and 84.2% in SiHa cells, and by 37.5%, 79.4%, and 87.8% in CaSki cells when cells were treated with 10, 20, and 40 µM of fisetin, respectively (Figure 2A). Western blot analysis was performed to examine the protein expression of uPA in cervical cancer cells. Fisetin inhibited the protein expression of uPA in a dose-dependent manner, compared to the control group in both two cervical cancer lines tested (Figure 2B). In addition, to verify the down-regulation of uPA, immunofluorescent labeling was performed. The bright red fluorescence from uPA in the control cells shows a constitutive expression of uPA, whereas 40 µM of fisetin significantly decreased uPA protein expression (Figure 2C). These results indicate that fisetin inhibited both the activity and protein of uPA in cervical cancer cells. To further investigate whether the inhibitory effect of fisetin on the activity and protein of uPA in cervical cancer cells was at the level of mRNA expression, a semi-quantitative RT-PCR analysis was performed. As shown in Figure 2D, after the treatment of fisetin for 48 h, the mRNA level of uPA also decreased significantly in a dose-dependent manner, compared to the control group, in both SiHa and CaSki cells. The fisetin-mediated change in the mRNA levels of uPA coincided with the protein levels, as indicated by the results from the Western blot analysis, suggesting that fisetin might regulate uPA expression at transcription levels. These findings suggest that the anti-metastatic effect of fisetin is related to the inhibition of uPA expression in cervical cancer cells.

### Fisetin Selectively Inhibits the Phosphorylation of p38 MAPK in Cervical Cancer Cells

**Figure 3:**
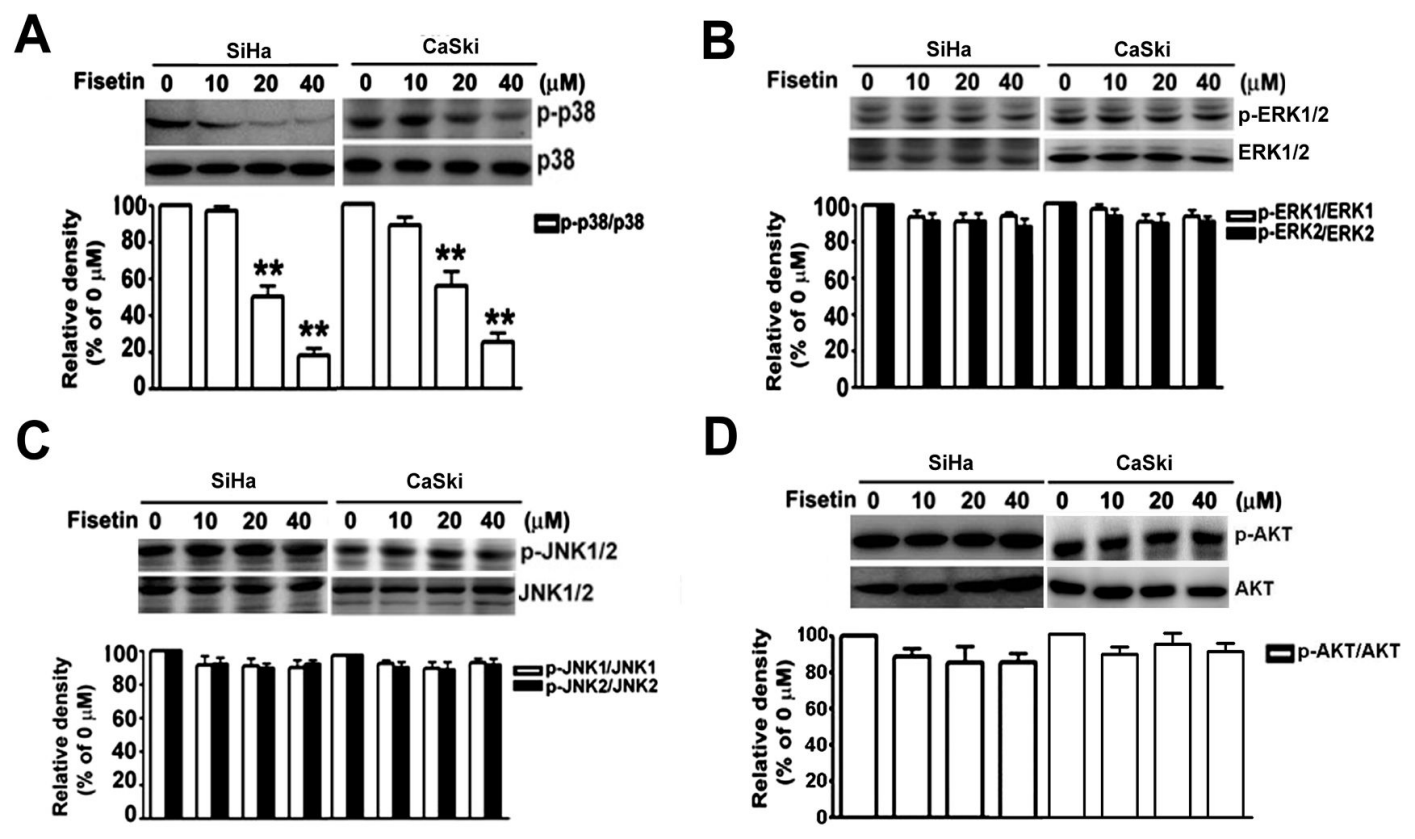
Effects of fisetin on the phosphorylation of MAPKs and AKT in cervical cancer cells. Cells were treated with the indicated concentrations of fisetin for 48 h. After treatment, cell lysate was extracted, and the phosphorylated (upper panel) and total amounts (lower panel) of (A) p38, (B) ERK1/2, (C) JNK1/2, and (D) AKT were determined by Western blotting using specific antibodies. The bottom plot show the relative density of the ratio of phosphorylated/total protein, compared to those of untreated cells from three independent experiments. Bars show the value as mean ± S.E. from three independent experiments. **, *P < 0.01*, compared with the untreated cells.

It has been reported that uPA is regulated by the MAPK or PI3K-Akt pathway in different types of cancers [37,38]. In this study, we further investigated which signaling pathway is critical in the fisetin-induced anti-metastatic effects in cervical cancer cells. Hence, AKT and MAPKs, including the extracellular signal regulated kinase (ERK), c-Jun N-terminal kinase (JNK), and p38 MAP kinase, were investigated after treatment of fisetin. Among all kinases tested, p38 MAPK phosphorylation was selectively and dramatically decreased in a dose-dependent manner without altering its protein amount in both SiHa and CaSki cells (Figure 3A), whereas the phosphorylation of ERK1/2 (Figure 3B), JNK1/2 (Figure 3C), and AKT (Figure 3D) showed no significant change, indicating that fisetin might repress the expression of uPA and reduce the migration and invasion of cervical cancer cells by inactivating the p38 MAPK pathway.

**Figure 4:**
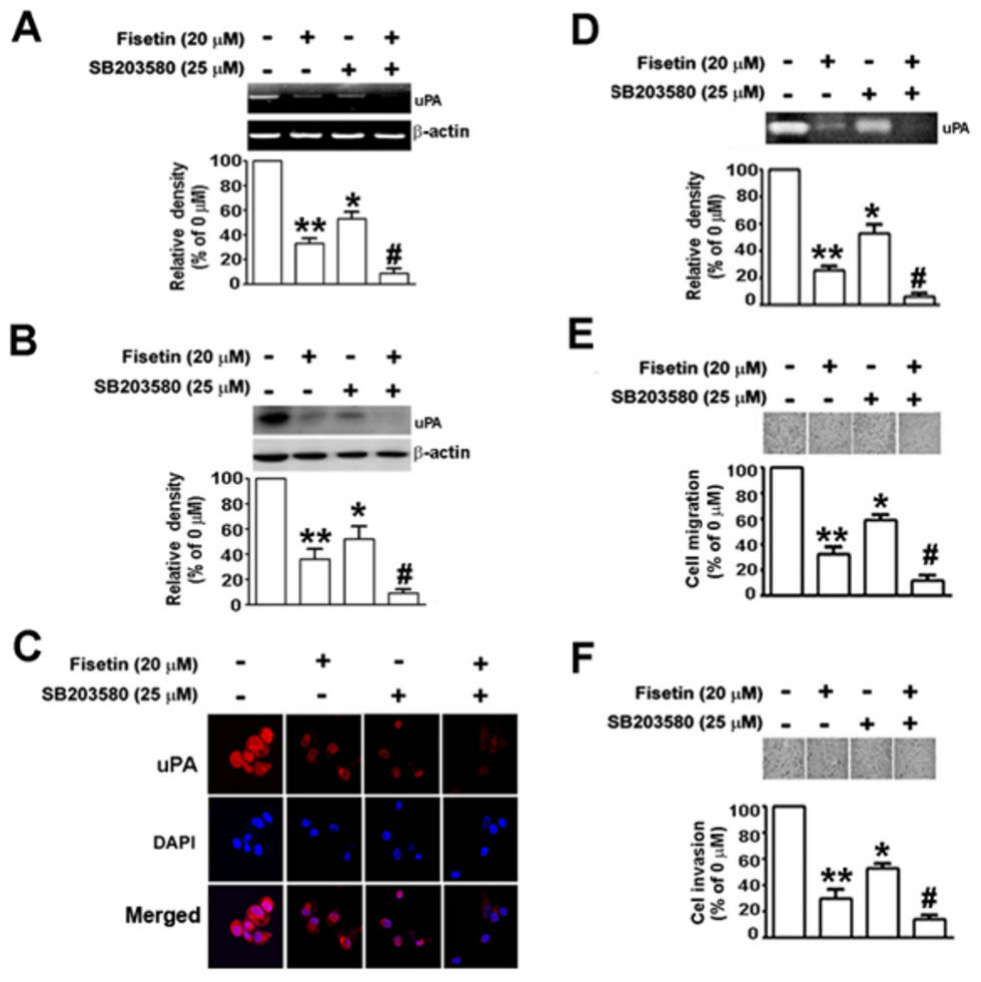
Effects of the inhibitor of p38 MAPK on fisetin-induced inhibition of uPA expression, cell migration and invasion in SiHa cells. Cells were pretreated with or without 25 µM of a p38 inhibitor, SB203580 for 2 h, and then treated with or without 20 µM of fisetin, as indicated, for another 48 h. (A) The mRNA level of uPA after each treatment was examined by RT-PCR. (B) The protein level of uPA was determined by Western blotting. β-actin was used as the internal control. (C) Cells were fixed, permeabilized, and immuno-stained with anti-uPA antibody (red) and cell nuclei were counter-stained with DAPI reagent (blue). (D) The conditioned medium from each treatment was collected, and the uPA activity was determined by casein zymography. The migratory (E) and invasive (F) ability of SiHa cells after treatment were determined. Migrating and invading cells were photographed using phase-contrast microscopy. Bars show the value as mean ± S.E. from three independent experiments. *, *P < 0.05*, untreated cells versus SB203580; **, *P < 0.01*; untreated cells versus fisetin; #, *P < 0.01*, fisetin versus SB203580 plus fisetin.

To further investigate whether the inhibition of uPA by fisetin occurred mainly through the inhibition of the p38 MAPK signaling pathway, SB203580, an inhibitor of p38 MAPK, was used for the treatment in the absence or presence of fisetin. As shown in Figure 4, both fisetin and SB203580 decreased the expression of uPA significantly in its mRNA level (Figure 4A) and protein level, as determined by Western blotting (Figure 4B) and by immunofluorescent staining (Figure 4C), and reduced its activity of secreted uPA in the conditioned medium (Figure 4D), and the inhibitory effects on above characters were significantly further enhanced by combined treatment of fisetin and SB203580 (the most right lane/bar in Figure 4A–4D). Taken together, these results suggest that fisetin acted on cervical cancer cells specifically through the p38 MAPK, but not the ERK1/2, JNK1/2 or AKT pathway. Moreover, in functional assays of anti-metastatic properties, SB203580 also facilitated the fisetin-induced cell migration (Figure 4E) and invasion (Figure 4F).

### Fisetin Suppresses the TPA-induced Phosphorylation of p38 MAPK and Expression and Secretion of uPA

**Figure 5:**
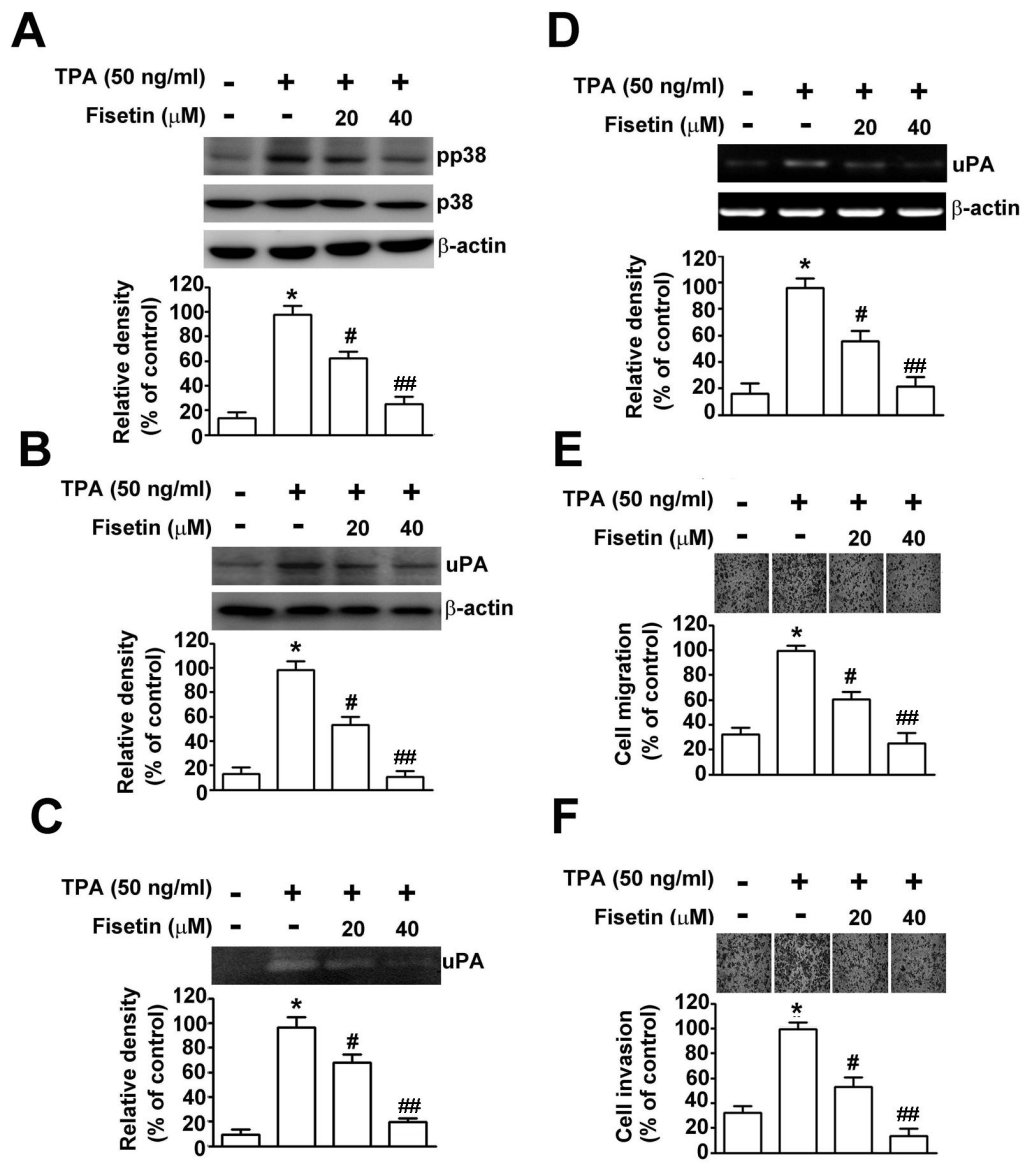
Effect of TPA on fisetin-induced inhibition of uPA expression, cell migration and invasion in SiHa cells. Cells were pretreated with or without fisetin for 2 h, and then treated with or without 50 ng/ml of TPA for 24 h. (A) The phosphorylated protein and total protein of p38 MAPK were determined by Western blotting. (B) The mRNA level of uPA after each treatment was examined by RT-PCR. (C) The protein level of uPA was determined by Western blotting. β-actin was used as the internal control. (D) The conditioned medium from each treatment was collected, and the uPA activity was determined by casein zymography. The migratory (E) and invasive (F) ability of SiHa cells after treatment were determined. Migrating and invading cells were photographed using phase-contrast microscopy. Values represent mean ± SD of three independent experiments. *, *P<0.01*, compared to untreated cells. # *P<0.05*, *# # P<0.01*, compared to the TPA treatment only.

Prior to investigating the pharmacological potential of fisetin on TPA-induced uPA expression, we asked whether fisetin inhibited the TPA-induced uPA expression in cervical cancer cells mainly by inhibiting the phosphorylation of p38 MAPK to suppress cell migration and invasion. We investigated the effect of fisetin on the phosphorylation of p38 MAPK in cells stimulated by 50 ng/ml TPA for 24 h. As shown in Figure 5A, fisetin inhibited the TPA-induced activation of p38 MAPK significantly in SiHa cells in a concentration-dependent manner. Fisetin inhibited TPA-induced uPA activity in a dose-dependent manner, as demonstrated by casein zymography (Figure 5B) and Western blot analysis (Figure 5C). To determine whether the inhibition of uPA secretion by fisetin was caused by a decrease in transcription, we performed RT-PCR. As shown in the semi-quantitative RT-PCR assay, treating SiHa cells with fisetin decreased the level of TPA-induced uPA mRNA expression (Figure 5D). Consistently, fisetin significantly decreased TPA enhanced the migratory (Figure 5E) and the invasiveness (Figure 5F) of SiHa cells. Taken together, these events indicate that the anti-metastatic properties of fisetin result from inactivating p38 MAPK, which represses the expression and activity of uPA in cervical cancer cells.

### Fisetin Inhibits the Transcriptional Activity of uPA by Disrupting Nuclear Translocation and Activity of NF-κB

**Figure 6:**
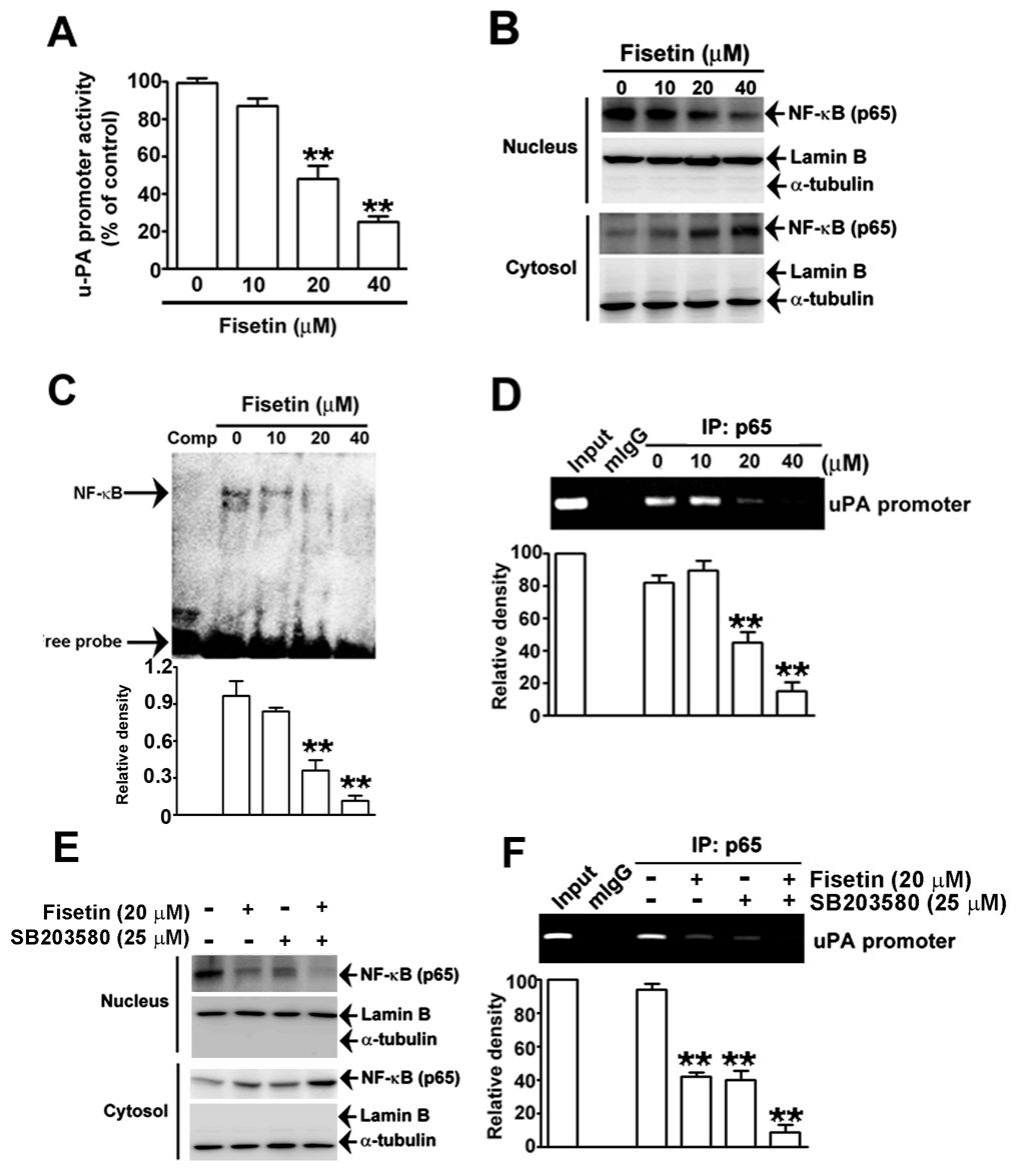
Effects of fisetin on uPA transcription and localization and DNA binding activity of NF-κB in SiHa cells. (A) Cells transfected with the luciferase reporter plasmid containing the promoter region of uPA were treated with increased concentrations of fisetin as indicated for 48 h. Cell lysate was extracted from each treatment, and the activity of uPA promoter was determined by luciferase activity. The plot showed the relative activity of the uPA promoter from three independent experiments. (B–D) Cells were treated with 0, 10, 20, or 40 µM of fisetin for 48 h. (B) Cell lysate was extracted as the nuclear and cytosolic fractions. The amount of NF-κB in each fraction was examined by Western blotting. Lamin B and α-tubulin were used as markers of nuclear and cytosolic fractions, respectively. (C) The nuclear extract from each treatment was incubated with biotin-labeled NF-κB-specific oligonucleotide with consensus sequence for NF-κB binding and underwent EMSA analysis to determine the DNA binding activity of NF-κB. The outer-left lane: competition was performed by addition of an unlabeled NF-κB oligonucleotide. Bands corresponding to NF-κB activity were quantified using densitometry and expressed in relative density (relative NF-κB activity) units comparing to that from untreated cells. (D) After treatment, proteins and chromatin within cells were cross-linked, and the DNA binding ability of NF-κB (p65) on the uPA gene promoter was determined by ChIP assay, as described in the materials and methods section. The localization of NF-κB (E) and its binding amount on uPA gene promoter (F) after treatment with or without fisetin and/or SB203580, respectively. The bottom plot shows the relative quantitative results, compared to that of the input. Bars show the value as mean ± S.E. from three independent experiments. **, *P < 0.01*, compared with the untreated cells.

NF-κB, a transcription factor, is known to be activated by p38 MAPK [39], and it translocates to the nucleus to regulate the expression of multiple genes involved in the invasion process [23]. Therefore, we investigated whether fisetin affects the nuclear translocation of NF-κB and influences the transcription of uPA. Results from the luciferase assay showed that fisetin significantly attenuated the promoter activity of uPA in a concentration-dependent manner (Figure 6A). The amount of NF-κB in the nucleus decreased, whereas its levels were elevated in the cytosol after treatment with fisetin (Figure 6B). To clarify the involvement of NF-κB transcription factor in the fisetin-induced down-regulation of uPA transcription, EMSA and ChIP assays were performed. As shown in Figure 6C, the DNA binding activity of NF-κB was inhibited significantly by up to 90.3% after treatment with fisetin in a concentration-dependent manner. Specifically, the binding capability of NF-κB on the promoter of uPA gene was repressed after treatment with fisetin at 20 and 40 µM in SiHa cells (Figure 6D). To further validate that fisetin-mediated repression of NF-κB nuclear translocation and its binding amount on uPA promoter is through p38 MAPK pathway, SB203580 was used to inactivate p38 activity, Inactivation of p38 or treatment of fisetin resulted in a decrease of NF-κB nuclear translocation (Figure 6E) and its binding amount on uPA promoter (Figure 6F), and these suppressive effects could be further enhanced by combined treatment of fisetin and SB203580 (Figure 6E and 6F). These results suggest that fisetin inhibits the nuclear translocation of the transcription factor NF-κB and reduces its binding amounts on the promoter of uPA, thereby repressing the transcription of uPA through p38 MAPK signaling pathway in cervical cancer cells.

**Figure 7:**
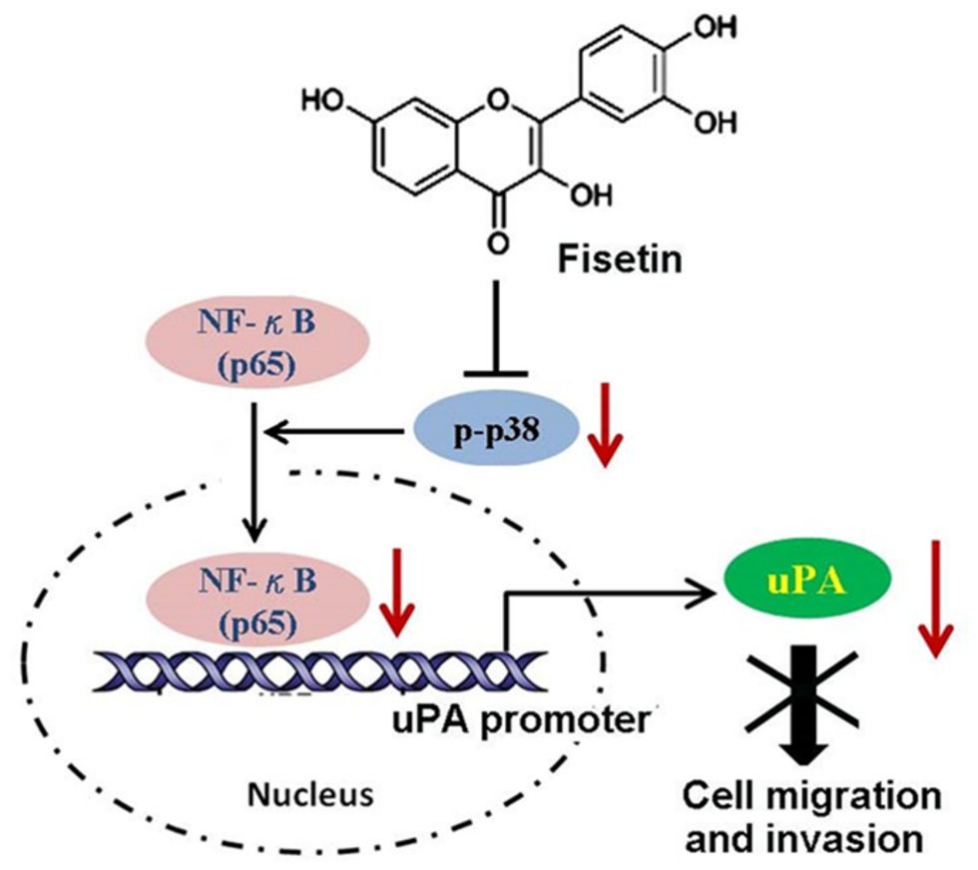
The proposed fisetin model in inhibiting the migration and invasion of cervical cancer cells. Fisetin inhibits the phosphorylation of p38 MAPK and impairs translocation of NF-κB to the nucleus. The decreased NF-κB in the nucleus reduces its binding on the promoter of the uPA gene, and results in repressing the expression and activity of uPA, thereby disrupting the migratory and invasive ability of cervical cancer SiHa cells.

We uncovered the anti-metastatic potential of fisetin in cervical cancer cells and elucidated its molecular mechanism, that is fisetin inhibits the activation of p38 MAPK, impairs translocation of NF-κB to the nucleus, and then decreases its binding amounts on the promoter of uPA gene, and results in repressing the expression and activity of uPA, leading to disrupting the invasiveness and motility of cervical cancer cells (Figure 7).

## Discussion

Most cancer deaths occur as a result of metastasis rather than the original tumor; therefore, inhibiting cancer-cell metastasis is a crucial aspect of cancer prevention. The objective of this study was to investigate whether the migration and invasion of cervical cancer cells could be regulated by fisetin and if that was the case, which molecular mechanisms and signaling pathways were involved. We elucidated the underlying mechanisms by which fisetin attenuates the migration and invasion of cervical cancer cells. We demonstrated that fisetin decreases migration and invasion in cervical cancer cells possibly by inactivating the p38 MAPK signaling pathway, inhibiting the nuclear translocation and DNA binding activity of NF-κB, and reducing the level of uPA expression, as well as having an anti-metastatic potential. Fisetin is a nontoxic dietary flavonoid that has been shown to possess anti-tumor properties. It therefore poses special interest in the development of a chemopreventive and/or chemotherapeutic agent for cancer.

Tumor cells are characterized as malignant cells that invade underlying connective tissues and migrate to form metastases at distant sites. This process includes disrupting the interaction between cells and the ECM. Therefore, identifying new dietary botanicals that have the capability to inhibit MMPs or uPA synthesis as well as cancer-cell invasion and migration is a significant issue. Several reports have shown that uPA related to the degradation of the matrix is required for tumor-cell metastasis and that an increased production of uPA correlates with the migration, invasion, metastasis, and angiogenesis of the tumor cells [40,41]. Elevated levels of uPA in cancers are a related prognostic marker in multiple types of cancers [42]. It is reported that uPA overexpresses in cervical cancer and plays a significant role in invasion and metastasis during advanced stages of cervical carcinoma [36,43]. Some studies have demonstrated that the anti-metastatic effect of flavonoid-rich plants is linked to uPA activity. For example, anthocyanins, which is major compound found in fruits, inhibited cell migration and invasion by down-regulating uPA expression in a variety of tumor cells [44]. EGCG (Epigallocatechin-3-gallate) from green tea, suppressed gliomas and oral cancer cell invasion by inhibiting uPA production [45,46]. The inhibitory effects of quercetin and baicalein on the migration and invasion of prostate cancer and liver cancer cells were related to the inhibition of uPA activity [47,48]. Consistent with these findings, results from this study demonstrate the anti-metastastic properties of fisetin on inhibition of invasion and migration in cervical cancer cells are due to suppression of uPA expression.

The up-regulation of uPA expression involves multiple signaling cascades, especially the MAPK and AKT pathways [49]. Based on this study, we speculated that fisetin may affect the MAPK signaling pathway, which has been reported to be critical for inhibiting migration and invasion during a variety of stress responses in several tumor cells. Our results showed that fisetin treatment inhibited the activation of p38 MAPK in a concentration-dependent manner, but had no effect on the alternative ERK1/2, JNK1/2, and AKT pathways. Furthermore, pretreatment of the p38 MAPK inhibitor, SB203580, significantly attenuated the migratory and invasive abilities in cervical cancer cells, which suggests that p38 MAPK is involved in regulation of migration and invasion signaling in this cancer type. Activation of p38 MAPK has been shown in various systems to be the mechanism for promoting the production of uPA, which is crucial for cell proliferation, migration, and invasion [50]. A previous study has suggested that the p38 MAPK pathway participates in invasive breast-cancer cell migration by regulating uPA expression [51]. Another report indicated that fisetin inhibits the migration and invasion of human lung cancer A549 cells by decreasing MMP-2 and uPA expression through inactivating the ERK1/2 signaling pathway [22]. Moreover, fisetin also inhibited the metastatic ability of PC-3 cells by reducing MMP-2 and MMP-9 expression through the suppression of the JNK1/2 and PI3K/Akt signaling pathways [23]. In this study, uPA was involved in the fisetin-induced inhibition of cell migration and invasion. Conversely, neither MMP-2 nor MMP-9 was involved (data not shown). Our findings differ from those in the previous studies, which may have been caused by cell-type specificity and the different cell invasion signaling pathways involved. Further investigation of the detailed mechanisms among p38 MAPK and the uPA in fisetin-inhibited cell migration and invasion may contribute to knowledge of the metastasis network. These results demonstrate the potential of fisetin as a potent chemotherapeutic drug against human cervical cancer through its inactivation of the p38 MAPK and uPA signaling pathways.

MAPKs participate in regulation of many biological functions, including cell survival, proliferation, and invasion. Accumulating evidences reveal that the roles of MAPKs in these functions are controversial and complicated, that depend on the stimuli, intensity, and duration, as well as cell types [52]. For instance, under stimulation of wound healing, p38 and ERK1/2 coordinate the dynamics of the processes through inducing migration by p38 and enhancing proliferation by ERK1/2 activation in corneal epithelial cells [53]. JNK plays opposite roles in carcinogenesis, which is involved in induction of apoptosis, but also implicated in promotion of cell survival and proliferation [54]. Recently, the dual role of p38 in response to different stimulations has been demonstrated in fibroblast cells. Exposure to anisomycin causes cellular stress and induces strong and persistent p38 activation, leading to cell cycle arrest. In contrast, mitogenic stimulation by serum results in weaker and transient p38 activation to induce cyclin D1 and inactivate Rb by phosphorylation, leading to enhancing proliferation [55]. In our current study, we found that fisetin inhibits the activity of p38 to reduce invasion and migration through down-regulation of uPA without significantly altering proliferation in cervical cancer cells, suggesting that the roles of fisetin-regulated p38 might be dominant in aggressive phenotypes (invasion and migration) rather than proliferation in cervical cancer cells. The roles of p38 in invasion and metastasis have been demonstrated [56]. Similarly, inactivation of p38 to reduce cell invasion through inactivation of MMPs by different stimuli have been reported in MCF-7 breast cancer cells by melatonin [57] or by pterostilbene [58], in MDA-MB-435 breast cancer cells by DT-13 [59], and in A549 lung adenocarcinoma cells by propofol [60].

Activation of NF-κB is crucial for mediating cancer-cell motility and invasion [61]. Constitutive NF-κB activation in cervical, breast, and glioma cancers has been demonstrated to be correlated with tumor progression and aggression as well as poor prognoses [62,63]. NF-κB activation occurs as it is transported from the cytoplasm to the nucleus when the inhibitory subunit is degraded. In the nucleus, NF-κB binds to the cognate sequence in the promoter region of many target genes. Therefore, the binding activity of NF-κB on the specific DNA region is a hallmark for its activation. Several studies have shown that inhibiting NF-κB activity can suppress cell migration, invasion, angiogenesis, and metastasis by inhibiting the expression of NF-κB downstream target genes, such as VEGF [64], uPA [65], MMP-9 [66], and CXCR4 [67]. Likewise, in this study, we observed that the translocation of NF-κB from the cytoplasm to the nucleus was inhibited by fisetin, and treating SiHa cells with fisetin resulted in the inhibition the DNA binding activity of NF-κB, as well as the NF-κB -dependent transcriptional activity of uPA in a dose-dependent manner (Figure 6). These results are comparable with those for MMP-9, MMP-2 and uPA expressions, and they are also in concord with previous reports on the inhibition of NF-κB by fisetin in prostate and lung cancer cells [22,37]

This study therefore provides insight into the way in which fisetin modulates the aggressive phenotype. Figure 7 shows the mechanism, that fisetin inhibits the migration and invasion in human cervical cancer cells. Suppression of uPA-dependent increase of cell migration and invasion by fisetin, at least in part, via suppression of p38 MAPK activation through reducing translocation to the nucleus and DNA-binding activities of NF-κB, leading to down-regulation of uPA expression. Future studies on fisetin may incorporate animal models to determine its efficacy in preventing migration and invasion in cervical cancer. Findings and observations from this study provide a crucial basis for further exploring the mechanisms of fisetin and its potential for preventing tumor metastasis, and its possibility as an anticancer agent or an adjunct to current cancer therapies.
